# Optimising head and neck cancer patient management: the crucial contributions of multidisciplinary tumour board decision-making

**DOI:** 10.3332/ecancer.2024.1710

**Published:** 2024-06-04

**Authors:** Bushra Ayub, Fizza Asif Qureshi, Nabeel Humayun Hassan, Fatima Shaukat, Talha Ahmed Qureshi

**Affiliations:** 1 Department of Learning Research Centre, Patel Hospital, Karachi 75300, Pakistan; 2 Department of Otolaryngology and Head and Neck Surgery, Patel Hospital, Karachi 75300, Pakistan; 3 Cyberknife and Tomotherapy Centre, Jinnah Postgraduate Medical Centre, Karachi 75510, Pakistan

**Keywords:** multidisciplinary team, squamous cell carcinoma, multidisciplinary tumour board, head and neck cancer, treatment plan

## Abstract

**Introduction:**

Squamous cell carcinoma (SCC) of the head and neck is a great burden globally, which is being tackled through treatment options of surgery, radiation therapy, chemotherapy, or a combination of these, to avoid disease-related mortality. Multidisciplinary tumour boards play a pivotal role in customising and deciding management plan based on clinical aspects. The objective of the study is to determine the concordance of opinion between the treatment plan of a primary physician and board members.

**Material and methods:**

This is a retrospective cross-sectional study that includes 137 head and neck carcinoma cases. They were discussed in the multidisciplinary tumour board meeting and were reviewed; all demographics were analysed including the tumour staging and the decisions of the primary physician was compared with those of the board. To check the concordance between primary surgeon plans or after board discussion Kappa agreement test was used.

**Results:**

Total of 137 patients were included in the study out of which 63 cases were pre-treatment and 74 cases were post-treatment, i.e., surgically treated cases, with the distribution being 46% and 54%, respectively. Most cases, totaling 120, were SCC, accounting for 80% of the total cases. Among the pre-treatment cases, T4a and N0 were the most common categories, with 29 and 40 cases, respectively. Similarly, in post-treatment cases, the majority fell into the T4a and N1 categories, with 29 and 38 cases, respectively. When comparing the primary surgeon's plan with the tumour board meeting decision, the agreement showed a value of 0.273, indicating a slight level of agreement between the two entities.

**Conclusion:**

Our data indicates that the multidisciplinary head and neck tumour board may have influenced the treatment plans of the primary surgeon, in approximately one in two patients (43.06%).

## Background

Squamous cell carcinoma (SCC) of the oral cavity is a globally frequent disease with a 2% incidence and 1.8% mortality rate estimated in 2020 [1], and it has a high recurrence rate. As a result, there is plenty of potential for researchers to pinpoint the factors that can lead to better results [2, 3]. We have seen that the diagnostic and therapeutic options for cancer have been changing in recent years due to ongoing advances in the field. Besides, in developing country like Pakistan, cancer management has challenges like cost and availability of resources. Therefore, multidisciplinary tumour boards (MDTBs) have become the standard practice for treatment decision making in oncology because of the growing importance of medical-surgical collaboration, particularly in tumours of the head and neck [4]. The collaboration of radiation oncology, medical oncology and head and neck surgery is necessary for head and neck oncology treatment decisions, which can be challenging and complex. An MDTB makes this coordination possible [5].

In our head and neck specialty MDTB, to ensure that each patient receives the best possible care, physicians must always consider all aspects of the patients’ present clinical status, including overall health, the specifics of their disease, co morbid diseases, current and prior therapies and their preferences, as well as consider local challenges like affordability and access to treatment. We debate and decide the most effective way to implement the necessary standard of care. A study showed that in comparison with stage IV head and neck cancer cases with and without MDTB discussion, it was reported that the 5-year survival rate improved [6]. Moreover, the discussions increased professional collaboration and minimised the amount of time needed before starting a treatment, particularly for multimodal treatments. The recommended treatment was also more readily accepted by patients who were present at board meetings [7].

Although tumour boards are thought to be crucial in the therapy of head and neck tumours, there are no studies that have looked at the function of multidisciplinary team (MDT) meetings in the treatment of head and neck tumour types. Therefore, it is unclear which patients will gain the most from the MDTB discussion. The effects of this specific viewpoint might result in a more effective use of the resources available to MDTB teams in a longer way, with a clearer concentration on the patients who stand to gain the most [8]. We believe that incorporation of tumour board in the management of head and neck cancer will benefit patients significantly and the impact of MDBT in establishing a final approach to treatment. The aim of the study is to determine the concordance of opinion between treatment plan of a primary physician and board members.

## Methods

This was a retrospective cross-sectional study, conducted in the department of Otolaryngology head and neck surgery monthly MDTB meeting at Patel hospital, tertiary care hospital located in Karachi, Pakistan. The study was approved from the institutional ethical review committee having ‘ERC# PH/IRB/2023/016’. The study's inclusion criteria encompassed all patients discussed during the MDTB meetings, while patients with benign diseases and those lacking adequate medical records were excluded from the study.

The MDTB meetings brought together a panel of specialists, including head and neck surgeons, medical oncologists, radiologists, radiation oncologists, as well as swallowing and speech therapists. The record of all the patients discussed in the MDTB meeting from January to December 2021 was evaluated. Demographic information of the patients, their diagnoses, and various parameters related to the primary disease, including tumour type, lesion site and pre- and post-T and N staging, were meticulously recorded. Additionally, the study documented the experience of the primary surgeons and the treatment plans proposed by the primary physician and the consensus made during the MDTB meetings.

The statistical analysis was performed by using SPSS version 21. Descriptive statistics was used to summarise the data as frequencies or percentages for qualitative variables. Chi-square was applied to see the association between the experience of the primary surgeon plan and with change in the treatment plan. To check the concordance between primary surgeon plans or after board discussion Cohen’s Kappa agreement test was used. A *p*-value <0.05 was considered statistically significant.

## Results

A total of 156 patients were discussed in the MDTB meeting. Out of which 137 cases met the inclusion/exclusion criteria and were included in the study. Out of which 63 were pre-treatment cases and 74 were post-treatment, i.e., surgically treated cases (46% versus 54%).

Many different types of tumours were discussed, the highest of which was 120 cases of SCC (80%). There were only three diagnosed cases of adenoid cystic carcinoma and mucoepidermoid carcinoma each, and two cases of lymphoma. Of the discussed cases, most were from the oral cavity region (99 cases, 72%). There were 5 cases of oropharynx, 12 cases of larynx, 6 cases of nose and paranasal sinuses, 3 cases of skin, 8 cases of parotid, 3 cases of nodal disease and 1 case of ear-related neoplasms.

**Figure 1.**
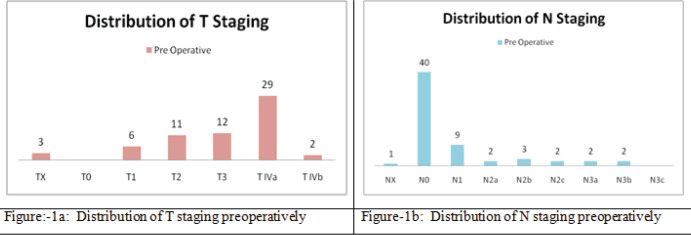
Distribution of tumour and nodal staging pre-operatively. (a): Distribution of T staging preoperatively. (b): Distribution of N staging preoperatively.

Regarding the staging of the primary tumour and nodal disease in the pre-treatment cases that were discussed, it was observed that there were three cases for which the primary tumour assessment could not be determined, but there were no cases with a T0 staging. Out of the discussed cases, there were 13 cases staged as T1, followed by 11 cases at T2. Additionally, there were 12 cases at the T3 stage. Notably, the highest numbers of cases, specifically 29 of them, were classified as T4a. Finally, only 2 cases were staged as T4b in the pre-treatment group (Figure 1a and b).

The highest numbers of cases were categorised into the nodal stages of N0, N1 and N2b. Specifically; there were 40 cases in which no nodal disease was detected in the pre-treatment assessment. Following this, there were nine pre-treatment cases with N1 disease, and three pre-treatment cases with N2b disease. The remaining nodal stage categories, N2a, N2c and N3a, each had two cases. Notably, there were no cases with N3b nodal disease, and only one case in which nodal disease assessment could not be determined.

**Figure 2.**
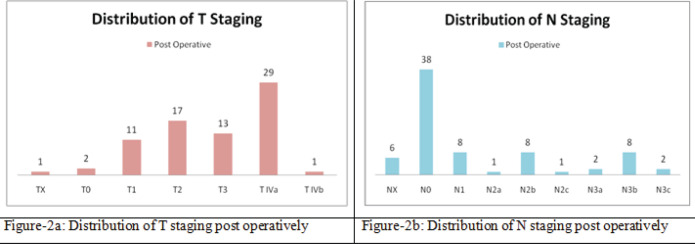
Distribution of tumour and nodal staging pre-operatively. (a): Distribution of T staging post operatively. (b): Distribution of N staging post operatively.

Out of the 74 post-treatment cases, demonstrated in Figure 2a and b, discussed in our MDTB, 29 patients (39%) were diagnosed T4a disease on final histopathology reporting, followed by 17 T2 staged, 13 T3 staged and 11 T1 staged cases, graphically represented in Figure 2a. Only one case each fell under the stage T4b and tumour that could not be assessed. There were also two such cases in which there was no primary site tumour, i.e., T0.

On post-treatment, i.e., post-operative histopathology report, also shown in Figure 2b, a total of 38 patients were diagnosed as having N0 disease, followed by 8 cases each of N1 and N2b. Then, only a handful of cases remained in the rest of the N-staging, i.e., six cases in which nodal disease could not be assessed, two cases each of the stage of N3a and N3b, and one case each diagnosed with N2a and N2c nodal disease.

Moreover, based on our data, there was a 33% chance that a surgeon with over 5 years of expertise would modify their treatment plan following an MDTB. However, Surgeons with less than 5 years of work experience had a 67% chance of changing their treatment plan. Hence there was a significant association with *p*-value <0.000.

Upon comparing the primary surgeon's plan with the decision made during the tumour board meeting, there has been a change in treatment plan after the MDTB as there was a slight concordance (0.273) which means that there was a significant change among decisions of two parties. Additionally, analysing the treatment plans before and after the MDTB revealed that 43.06% of primary plans were altered after the meeting, while 56.93% remained unchanged and proceeded with the initially proposed plan, as detailed in Table 1. No notable distinction was observed when comparing the decisions for early-stage and advanced-stage tumours Table 2. Consequently, irrespective of the tumour stage being early or advanced, it is imperative to discuss all patients in the MDBT meeting.

## Discussion

Globally, South Asia has been identified as having the highest reported incidence of oral cancer [1]. Over the past century, concerted efforts have been made to establish tumour boards specialised in specific anatomical sites in countries such as Pakistan and other South Asian Association for Regional Cooperation nations. These clinical meetings have demonstrated a significant impact on the improvement of cancer-related healthcare [9, 10].

In Western regions, such as England and North America, the development of patient-centered MDTB meetings has evolved significantly over time and is now a mandatory practice [5]. However, in countries such as Pakistan and India, clinicians have been somewhat slow to adopt this transformative shift in their clinical practice [11]. MDTs not only improve patient care but also provide physicians with valuable insights into the real-life challenges patients face in their pursuit of better healthcare. Moreover, there is a low level of awareness regarding tumour boards among undergraduate students, and this can be rectified by incorporating additional awareness sessions and credit hours into their academic curriculum. Such measures will contribute to shaping a future generation of oncologists and surgeons in Pakistan who possess a deeper understanding of tumour boards [12–14].

The first site-specific head and neck tumour board was established in 2007 at Aga Khan University Hospital in Karachi. While General tumour board meetings have been a longstanding practice at our institute, the head and neck tumour board was introduced more recently in 2021, coinciding with the COVID-19 pandemic. Presently, Karachi hosts four regularly convened head and neck tumour board meetings across various institutions. However, considering that at least ten institutes in the city routinely manage head and neck cancer patients, there remains a palpable need to raise awareness among clinicians about the significance of this aspect of cancer management. Since 2010, Karachi has been hosting a fortnightly event called the City Tumour Board, which aims to support hospitals lacking their own tumour boards. Esteemed oncologists and surgeons voluntarily convene on alternate Sundays to discuss cases, reach a consensus and provide valuable assistance to tertiary care centers without tumour boards [15].

Our analysis of cases discussed in 2021 reveals a predominant occurrence of oral SCC, consistent with findings in other studies [16, 17]. Notably, a substantial 90% of the study population presented with advanced-stage cancer, which mirrors the typical scenario in South Asia, where patients often seek medical attention at advanced stages, as corroborated by regional studies [18, 19]. Notably, our data highlights that pre-operative imaging struggled to accurately identify extra capsular disease spread, a divergence from Western literature [20–22]. This underscores the imperative for meticulous evaluation of extra capsular spread through collaborative discussions among clinicians and radiologists before initiating treatment.

There was a local study reported that in 70% of cases initial treatment strategy changed after a tumour board meeting [15]. Also, our data indicates that the multidisciplinary head and neck tumour board influenced treatment plans in approximately one in two patients (43.06%). These results align with other international literature, which include Kurpad *et al* [23] who reported a 38% change in diagnosis or treatment following multidisciplinary meetings for urologic malignancies, with most changes involving treatment plan modifications. Despite some reluctance among clinicians to attend these meetings due to the perceived efficacy of their own treatment plans, our study presents a different perspective. The Kappa coefficient calculated in our study revealed slight agreement, suggesting that the consensus opinion of the tumour board often differed from the primary physician's plan. This aligns with findings presented by Wheless *et al* [8] and Kurpad *et al* [23], emphasising the importance of pre-treatment case discussions in shaping appropriate treatment plans.

One intriguing observation made was the increased likelihood of treatment plan modification among surgeons with less than 5 years of experience compared to those with over 5 years of experience 67% versus 33%. While we did not uncover similar findings in the existing literature, there are reports suggesting that a surgeon's experience can impact various factors. For instance, Maruthappu M *et al* [24] concluded in their systematic review, albeit not specifically focused on surgical oncologists, that surgical performance and outcomes are directly linked to a surgeon's experience. Similarly, Lee *et al* [25] investigated the correlation between surgeon volume and treatment costs, finding that treatment costs tend to be lower when handled by surgeons with a high volume of cases, indicating that increased experience likely accompanies higher case volumes. However, we were unable to find any literature regarding differences in treatment plans based on experience, specifically in the context of oral cancer treatment, making this a unique finding in our report. It is important to acknowledge that this study has several potential limitations. Firstly, it was unable to identify the reasons behind any discrepancies between the primary physician's plan and the tumour board's plan. Additionally, the study did not assess adherence to treatment guidelines. Another notable limitation is that the study solely evaluated diagnostic and treatment decisions, without considering actual patient outcomes. Furthermore, no comparison was made with a non tumour board group, and it is unlikely that a randomised trial will be conducted to measure these outcomes. While it is anticipated that a multidisciplinary approach results in improved decisions, better treatments and ultimately superior outcomes, this analysis does not allow us to conclude that the MDTB enhances cancer survival.

Moreover, it is worth mentioning that the findings of this study may have limited generalisability because of the fact that our institution is a comprehensive cancer center with a sizable population of head and neck tumour patients. The success of our MDTB meetings stems from the expertise contributed by various fields such as otolaryngology, neurosurgery, plastic surgery, medical oncology, radiation oncology, pathology, radiology, dental, oral, maxillofacial surgery and social work, all of which specialise in addressing issues related to head and neck cancer. It remains uncertain how closely the outcomes of this study can be applied to multidisciplinary conferences held at smaller academic or community programs.

It is plausible that the use of teleconferencing for communication could provide an opportunity for these smaller centers to collaborate with larger groups. However, it is important to note that none of the patients included in the current study were teleconference patients.

## Conclusion

To conclude, our MDTB meetings influence the primary surgeon plans for one in every two cases that are discussed, which leads us to believe that these meetings are a major step in deciding the better treatment option for our patients. Furthermore, the need of discussion of early-stage cancers is the same as of the advanced stage cases, for better treatment insight and patient case.

## List of abbreviations

MDTB, Multidisciplinary tumor board; MDTs, Multidisciplinary teams; SCC, Squamous cell carcinoma.

## Conflicts of interest

The authors declare in this study, there were no conflict of interest.

## Funding

There was no funding raised for this study.

## Authors’ contributions

Bushra Ayub: Wrote the initial draft, study design, data entry or analysis, drafting the final work and reviewed critically.

Fizza Asif Qureshi: Wrote the initial draft, study design, data entry or interpretation, drafting the final work and reviewed critically.

Nabeel Humayun Hassan: Ensured all aspects of the work that questions related to the accuracy or integrity of any part of the work are appropriately investigated and resolved and reviewed critically for important contents.

Fatima Shaukat: Drafting the final manuscript and reviewed critically for important contents.

Talha Ahmed Qureshi: Wrote the intial draft, and reviewed critically for important contents.

## Figures and Tables

**Table 1.** Head and neck tumour outcomes.

Type of change in treatment plan from the tumour board presentation	All patients (*n* = 137)
No change in treatment plan	78 (56.93%)
Change in treatment plan	59 (43.06%)

**Table 2.** Final staging before and after MDBT.

Final staging	No change in treatment plan *N* (%)	Change in treatment plan *N* (%)	*p*-value
Early staged	19 (26%)	12 (21%)	0.545
Advanced staged	54 (74%)	44 (79%)	
